# Central Taurine Attenuates Hyperthermia and Isolation Stress Behaviors Augmented by Corticotropin-Releasing Factor with Modifying Brain Amino Acid Metabolism in Neonatal Chicks

**DOI:** 10.3390/metabo12010083

**Published:** 2022-01-16

**Authors:** Mohamed Z. Elhussiny, Phuong V. Tran, Yuriko Tsuru, Shogo Haraguchi, Elizabeth R. Gilbert, Mark A. Cline, Takashi Bungo, Mitsuhiro Furuse, Vishwajit S. Chowdhury

**Affiliations:** 1 Laboratory of Regulation in Metabolism and Behavior, Graduate School of Bioresource and Bioenvironmental Science, Kyushu University, Fukuoka 819-0395, Japan; mohamedzakaria@vet.aswu.edu.eg (M.Z.E.); tran.viet.phuong.737@m.kyushu-u.ac.jp (P.V.T.); turu.yuriko.508@s.kyushu-u.ac.jp (Y.T.); furuse@brs.kyushu-u.ac.jp (M.F.); 2 Department of Animal & Poultry Behavior and Management, Faculty of Veterinary Medicine, Aswan University, Aswan 81528, Egypt; 3 Department of Biochemistry, Showa University School of Medicine, Tokyo 142-8555, Japan; shogo@med.showa-u.ac.jp; 4 School of Neuroscience, Virginia Polytechnic Institute and State University, Blacksburg, VA 24061-0306, USA; egilbert@vt.edu (E.R.G.); macline2@vt.edu (M.A.C.); 5 Department of Bioresource Science, Graduate School of Biosphere Science, Hiroshima University, Higashi-Hiroshima 739-8528, Japan; bungo@hiroshima-u.ac.jp; 6 Division of Experimental Natural Science, Faculty of Arts and Science, Kyushu University, Fukuoka 819-0395, Japan

**Keywords:** taurine, CRF, chicks, isolation stress, sedation, hypnosis

## Abstract

The objective of this study was to determine the effects of centrally administered taurine on rectal temperature, behavioral responses and brain amino acid metabolism under isolation stress and the presence of co-injected corticotropin-releasing factor (CRF). Neonatal chicks were centrally injected with saline, 2.1 pmol of CRF, 2.5 μmol of taurine or both taurine and CRF. The results showed that CRF-induced hyperthermia was attenuated by co-injection with taurine. Taurine, alone or with CRF, significantly decreased the number of distress vocalizations and the time spent in active wakefulness, as well as increased the time spent in the sleeping posture, compared with the saline- and CRF-injected chicks. An amino acid chromatographic analysis revealed that diencephalic leucine, isoleucine, tyrosine, glutamate, asparagine, alanine, β-alanine, cystathionine and 3-methylhistidine were decreased in response to taurine alone or in combination with CRF. Central taurine, alone and when co-administered with CRF, decreased isoleucine, phenylalanine, tyrosine and cysteine, but increased glycine concentrations in the brainstem, compared with saline and CRF groups. The results collectively indicate that central taurine attenuated CRF-induced hyperthermia and stress behaviors in neonatal chicks, and the mechanism likely involves the repartitioning of amino acids to different metabolic pathways. In particular, brain leucine, isoleucine, cysteine, glutamate and glycine may be mobilized to cope with acute stressors.

## 1. Introduction

Stress is a state of altered body homeostasis that is mediated through physiological changes and behavioral responses. While the stress response is crucial for animal adaptation to a novel environment, it is linked to anxiety-related disorders when chronically activated [1], and, in particular, social isolation stress has been more prevalent in recent years due to the COVID-19 pandemic [2,3]. Chicks serve as a model animal for anxiety, and when isolated from their social group, display predictable, quantifiable behaviors [4] that have been described [5,6]. For instance, an isolation stress paradigm is useful for screening anxiolytic drugs in chicks with recordings of spontaneous activity and distress vocalizations [5,7,8]. 

Corticotropin-releasing factor (CRF), a 41 amino acid polypeptide that originates from the paraventricular nucleus of the hypothalamus, modulates stress-related effects in the central nervous system (CNS), such as alteration in wakefulness and sleep [9,10]. This peptide has a variety of biological effects and is an indicator of hypothalamic–pituitary–adrenal (HPA) axis activity [11,12,13]. Studies of the CRF effects in animal models involve the administration of CRF or receptor antagonists into the brain, usually into the ventricular system (i.e., intracerebroventricular; ICV). CRF increased the magnitude of anxiety among socially separated/isolated chicks [14,15,16] and was involved in stress-induced hyperthermia (SIH) in rats [11,17,18]. In general, across species, CRF treatment induces strong anorectic and thermogenic effects [19], and the effects in chicks, including behavioral responses to social isolation stress, have been described [20,21]. Thus, CRF is a key mediator of stress responses in birds and mammals, with effects on food intake, body temperature, and metabolism likely serving to alter or restore whole-body homeostasis.

Core body temperature is a key indicator of the physiological state during the response to a stressor. SIH is stimulated in response to the activation of the HPA axis [22], and social isolation-SIH occurred in rats [23] and humans [24]. SIH was used to screen for anxiolytic activity in singly housed mice, in which rectal temperature was elevated relative to the group-housed mice [25]. Thus, body temperature is a useful marker for anxiolytic activity and drug screening.

The brain amino acid concentrations in neonatal chicks are altered under stressful conditions, including social isolation stress and fasting stress [8]. Hamasu et al. [8] reported that diencephalic alanine, arginine, asparagine, aspartic acid, phenylalanine, proline and serine were reduced in response to isolation-induced stress. Several amino acids have sedative and hypnotic effects in chicks, including L-proline [8], L-serine [26], L-ornithine [16] and L-aspartate [6]. Thus, it stands to reason that the attenuation of stress-related behaviors could be achieved through nutritional intervention with dietary amino acids. Taurine (2-aminoethane sulfonic acid) is found in high concentrations in mammalian tissues, including the brain [27]. Taurine plays a role in multiple physiological functions, including metabolic activity, antioxidation, osmoregulation, membrane stabilization and neurotransmission [27,28,29]. Taurine, an inhibitory neurotransmitter in the brain, has a positive allosteric modulatory effect on ligand-gated chloride channels in neurons, including the ionotropic γ-amino butyric acid receptor (GABA_A_-R) and the glycine receptor, as well as inhibitory effects on other ligand- and voltage-gated cation channels, such as the *N*-methyl-*D*-aspartate receptor [30,31]. Our recent findings showed that the central injection of taurine caused hypothermia in a dose-dependent manner in chicks that was mediated through GABA_A_-R [32]. In addition, taurine was reported to induce anxiolytic effects in mice and rats [33,34], and the consumption of taurine-supplemented diets was associated with antidepressant-like behaviors in forced swimming-tested mice [35]. However, there is no report on the use of taurine to minimize social isolation stress and SIH.

The hypothalamus is considered to be the central hub for thermoregulation [36]. Kataoka et al. [37] reported that the rostral modulatory raphe and the dorsomedial hypothalamus mediated SIH. However, the locus coeruleus (LC), a small brainstem nucleus, is considered the primary site for norepinephrine production in the brain, which is implicated in the etiology of anxiety or stress [38,39]. Furthermore, LC dendrites receive input from excitatory terminals containing CRF [40]. 

In this study, we assessed the effects of centrally administered taurine and CRF on rectal temperature and behavioral responses in a neonatal chick social isolation stress model. We also investigated the involvement of amino acids with a focus on the hypothalamus and brainstem, which contain nuclei that are known to be involved in regulating the stress response and SIH.

## 2. Results

### 2.1. Changes in Rectal Temperature after Social Isolation and ICV Injection of Taurine and CRF 

**Figure 1:**
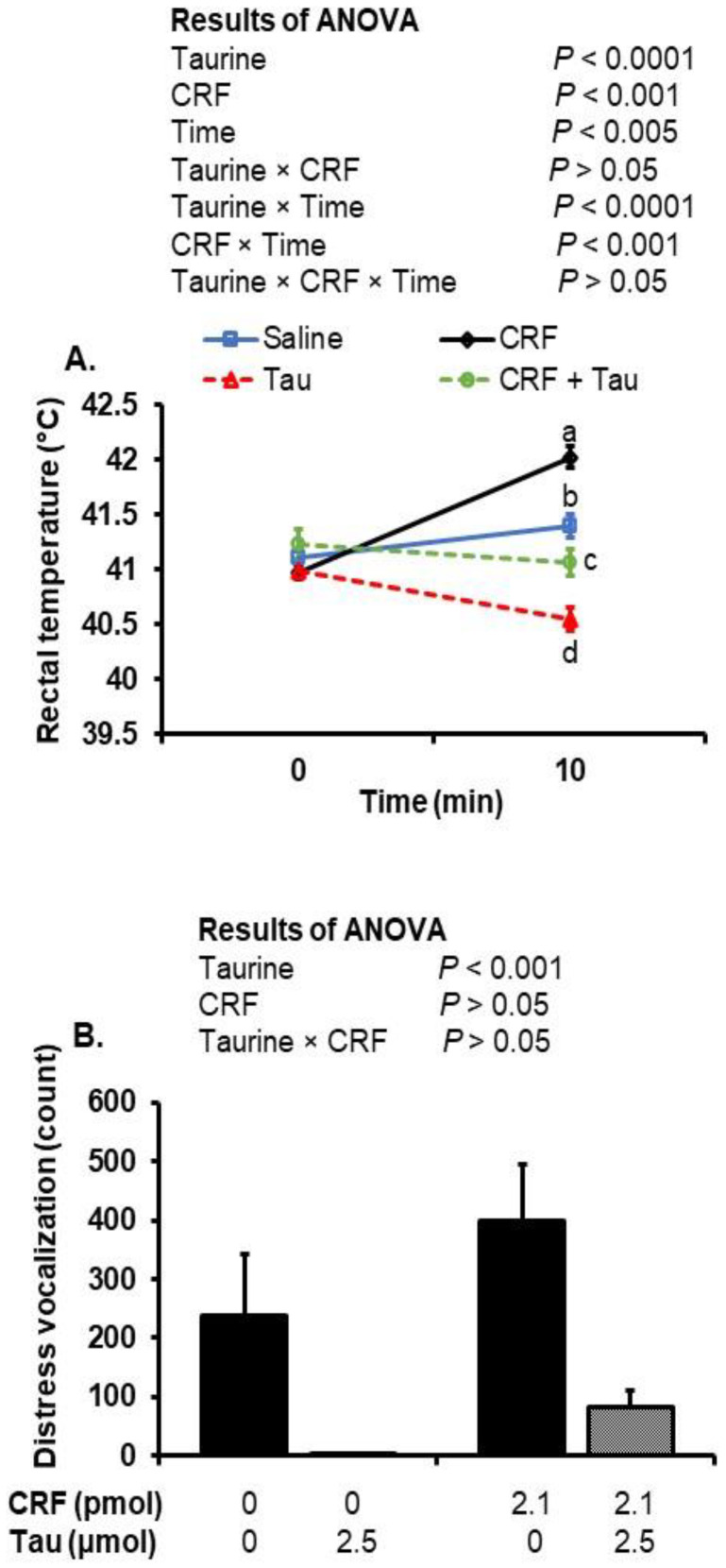
Rectal temperature (**A**) and distress vocalization (**B**) of chicks following the ICV injection of saline (control), taurine (2.5 µmol), CRF (2.1 pmol) or CRF plus taurine under social isolation stress. Values are expressed as mean ± SEM from chick groups (8–10). Means with different superscripts indicate statistically significant differences (*p* < 0.05). Tau, taurine; CRF, corticotropin-releasing factor.

The central injection of taurine significantly (*p* < 0.0001) reduced rectal temperatures, whereas CRF significantly (*p* < 0.001) increased temperatures, compared to the saline-injected controls (Figure 1A). There were interactions between taurine and time (*p* < 0.0001) and CRF and time (*p* < 0.001), which demonstrate the hypo- and hyperthermia induced by taurine and CRF injections, respectively. When taurine and CRF were co-administered, there was no effect on temperature.

### 2.2. Changes in Distress Vocalizations after Social Isolation Stress and Injection of Taurine and CRF 

The injection of taurine with or without a co-injection of CRF was associated with a significant (*p* < 0.001) decrease in the number of distress vocalizations compared to the saline- and CRF-injected chicks (Figure 1B). 

### 2.3. Behavioral Alterations after Social Isolation Stress and Injection of Taurine and CRF 

**Figure 2:**
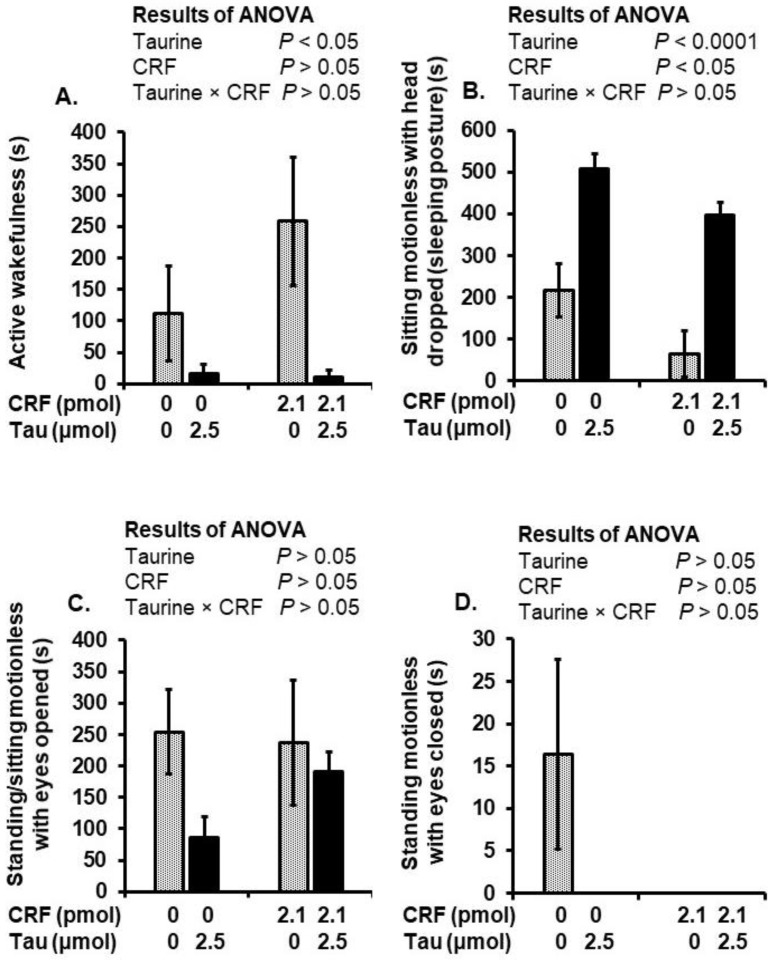
Active wakefulness (**A**), sitting motionless with the head dropped (sleeping posture) (**B**), standing/sitting motionless with eyes open (**C**) and standing motionless with the eyes closed (**D**) of the chicks following an ICV injection of saline (control), taurine (2.5 µmol), CRF (2.1 pmol) or CRF plus taurine under social isolation stress. Values are expressed as mean ± SEM from the chick groups (9–10). Means with different superscripts indicate statistically significant differences (*p* < 0.05). Tau, taurine; CRF, corticotropin-releasing factor.

The injection of taurine with or without CRF significantly (*p* < 0.05) decreased the time spent in active wakefulness compared to the saline- and CRF-injected chicks (Figure 2A). Compared to the CRF- and saline-injected chick groups, taurine significantly (*p* < 0.0001) increased the time spent in the sleeping posture when injected alone or with CRF. However, CRF significantly (*p* < 0.05) decreased the time spent in the sleeping posture, regardless of a co-injection with taurine, compared to the saline- and taurine-injected chick groups (Figure 2B). No significant changes were detected in the time spent standing or sitting motionless with the eyes opened or the time spent standing motionless with the eyes closed (Figure 2C,D).

### 2.4. Brain Amino Acid Changes after Social Isolation Stress and Injection of Taurine and CRF

Diencephalic leucine, isoleucine, tyrosine, glutamate, asparagine, alanine, β-alanine, cystathionine and 3-methylhistidine concentrations significantly (*p* < 0.05) decreased in taurine- and taurine–CRF-co-injected chicks compared with saline- and CRF-injected chicks (Table 1). Diencephalic taurine concentrations significantly (*p* < 0.0001) increased in taurine–CRF-co-injected chicks compared with saline- and CRF-injected chicks. The other diencephalic amino acid concentrations are shown in Supplementary Table S1. 

The brainstem concentrations of isoleucine, phenylalanine, tyrosine and cysteine significantly (*p* < 0.05) decreased in the taurine–CRF-co-injected chicks compared with the saline- and CRF-injected chicks (Table 2). The injection of taurine and co-injection with CRF significantly (*p* < 0.05) increased glycine concentrations. Taurine concentrations were significantly (*p* < 0.0005) elevated after the taurine and CRF co-injection. The interactions indicate that taurine and tyrosine were differentially affected after the injection of CRF or taurine. Other brainstem amino acid concentrations are shown in Supplementary Table S2.

## 3. Discussion

In this study, we investigated the effects of social isolation stress, central taurine and CRF on rectal temperature, behavioral responses and brain amino acid concentrations. The ICV injection of CRF caused hyperthermia, which was attenuated by a co-injection with taurine. CRF induces hyperthermia through the activation of the HPA axis [41] and the dose-dependently increased O_2_ consumption, CO_2_ production and heat production in the chicks [42]. On the other hand, the central injection of taurine induced dose-dependent hypothermia in chicks [32] and in rabbits [43]. It could be speculated that taurine-induced hypothermia and attenuated CRF magnified SIH through a reduction in heat production. Further study is needed to elucidate how taurine antagonizes CRF-induced hyperthermia, although we did observe changes in some behaviors and brain amino acids that may help explain such effects. For instance, central taurine decreased the number of distress vocalizations, decreased the time spent in active wakefulness and increased the time spent in the sleeping posture. Distress vocalization is considered to be an important feature of the stress response during isolation [5,44]. 

Glutamate, a non-essential amino acid, is locally synthetized from glutamine or Krebs cycle intermediates and acts as an excitatory neurotransmitter in the CNS [45]. Glutamate in the brain is made locally due to low permeability at the blood–brain barrier (BBB) [46]; a very small amount of glutamate can pass through the BBB under high blood glutamate concentrations [47]. In addition, leucine and isoleucine, branched-chain amino acids (BCAAs), are important sources of nitrogen for glutamate synthesis [48,49,50,51]. Leucine increased glutamate dehydrogenase and glutamate synthesis from different nitrogen sources in the liver of pigs [52]. Alanine donates its amino group to α-ketoglutarate by alanine transaminase to form glutamate [53]. Thus, decreased leucine, isoleucine and alanine concentrations may reflect their contribution to the synthesis of glutamate, which was decreased in the present study in response to the taurine injection. The reason for diminished glutamate could be connected to the synthesis of glutathione (GSH), a glutamate-containing tripeptide (γ-glutamyl-cysteine-glycine). Glutamate and cysteine are used as substrates by γ-Glu-Cys synthetase to produce γ-glutamyl-cysteine dipeptide (γ-Glu-Cys), which is then combined with glycine to produce GSH [54,55]. Central GSH-induced sedation and hypnosis in neonatal chicks [56]. Furthermore, Glu-Cys and Glu-Gly dipeptides [55] and glutamate [57] decreased distress vocalizations and the time spent in active wakefulness in a dose-dependent manner in neonatal chicks. Cystathionine is synthesized from homocysteine and serine and then metabolized to cysteine by cystathionine γ-lyase to form GSH [58]. Decreased brain glutamate, cystathionine and cysteine in taurine-injected chicks suggest that these amino acids may be utilized for GSH synthesis to induce sedation and hypnosis. ICV glycine produced sedative effects in neonatal chicks that were mediated by the glycine receptor [26]. Although it is difficult to hypothesize why glycine concentrations were increased in the current study, taurine may stimulate the glycine metabolism to induce sedation and hypnosis.

Tyrosine is the precursor to catecholamine biosynthesis in the brain. It is converted into L-DOPA via tyrosine hydroxylase and then into dopamine and norepinephrine through two sequential enzymatic reactions pre-synaptically [59,60]. Our recent study showed that a higher dose (5 µmol) of ICV taurine stimulated catecholamine biosynthesis in the regulation of body temperature in neonatal chicks (unpublished data). In the present study, taurine-injected chicks, irrespective of CRF injection, had less diencephalic and brainstem tyrosine. Thus, taurine may stimulate the tyrosine metabolism to produce catecholamines, which may be involved in the regulation of body temperature and stress response in chicks.

Taurine and β-alanine were reported to be antagonistic to each other at the BBB in terms of transport because they are both in the β-amino acid category [61]. The chronic supplementation of β-alanine decreased brain taurine concentrations [62]. Increased brain taurine could thereby lead to a decrease in β-alanine concentrations in the brain. 

In conclusion, the ICV injection of taurine attenuates CRF-induced hyperthermia and isolation stress behaviors in neonatal chicks. Brain leucine, isoleucine, glutamate, cysteine, cystathionine and glycine may be utilized in GSH synthesis to regulate the stress response through inducing sedative and hypnotic effects. These results suggest that taurine may serve as a novel isolation stress-relieving agent. Further study is needed to determine whether such effects are achievable through a dietary or other peripheral routes of administration.

## 4. Materials and Methods

The experimental procedures were conducted in accordance with the guidelines for animal experiments of the Faculty of Agriculture and the Graduate Course of Kyushu University and complied with Law No. 105 and Notification No. 6 of the Japanese government. The experimental protocol was approved by the animal experiment committee in Kyushu University (authorization no. A20-282-2).

### 4.1. Animals

Fertilized eggs (Julia layer strain, *Gallus gallus domesticus*) from a nearby hatchery (Tsuboi hatchery, Kumamoto, Japan) were incubated at 37.6 °C with 60% relative humidity in an incubator (Rcom Maru Deluxe MAX 380, Autoelex Co., Ltd., Gimhae-si, Korea) with auto-turning every hour until day 18. On embryonic day 7, the eggs were candled, and undeveloped and dead embryos were discarded. On embryonic day 19, the eggs were transferred to hatching trays. After hatching, day-old layer chicks were reared in groups with 20 birds per metal cage (floor space: 50 cm × 35 cm; height: 33 cm) under thermoneutral temperature (CT; 30 ± 1 °C) and continuous light. Feed (Adjust diet; Toyohashi Feed Co., Ltd., Aichi, Japan; metabolizable energy: >12.55 MJ/kg, protein > 23%) and water were provided *ad libitum*. On the second day after hatching, chicks were feather-sexed and male chicks were separated and used for the experiments. At four days old, chicks were assigned to treatment groups based on body weight. 

### 4.2. Injection Procedure

The taurine was purchased from Wako Pure Industries, Ltd. (Osaka, Japan). The rat CRF was purchased from Peptide Institute, Inc. (Osaka, Japan). The taurine and CRF were dissolved in 0.85% saline containing 0.1% Evans blue. Evans blue solution was injected into the control group chicks, as described [32,63]. The drugs were incubated on ice during the experiments. The ICV injection was performed at five days of age, using a microsyringe, as described by Davis et al. [64]. Briefly, the head of the chick was introduced into an acrylic device that provides an injection site guide, and the solutions were injected into the left lateral ventricle, and the syringe was held in place for 10 s to prevent overflow. The ICV injection procedure is not stressful for the chicks [65]. 

### 4.3. Experimental Design

On the day of the experiments, the chicks (five days old; *n* = 10) were injected with 10 µL of saline, CRF (2.1 pmol [6,16]), taurine (2.5 µmol [32]), or CRF plus taurine (2.1 pmol + 2.5 µmol). The chicks were then individually housed in an acrylic glass chamber (40 cm × 30 cm × 20 cm) at a temperature of 30 ± 1 °C, and water and diet were withheld. The vocalizations and behaviors were audio and video recorded from three different directions for 10 min using digital cameras (JVC, Everio, Japan). Behaviors were classified into four categories, including active wakefulness, standing/sitting motionless with the eyes open, standing motionless with the eyes closed and sitting motionless with the head dropped (sleeping posture) [66]. The vocalizations were counted using a digital hand tally counter, and the behaviors were analyzed by a researcher blind to treatment. A digital thermometer with a precision of ±0.1 °C (Thermalert TH-5, Physitemp Instruments Inc., Clifton, NJ, USA) was used to measure the rectal temperature at 0 and 10 min post-injection by inserting the thermistor probe into the rectum via the cloaca to a depth of approximately 2 cm, as we reported [32,67]. The chicks were anesthetized 10 min post-ICV injection with isoflurane (Mylan Pharmaceutical Co., Ltd., Tokyo, Japan) before the collection of blood samples from the jugular vein. The chick brains were dissected following euthanasia, and the diencephalons (thalamus and hypothalamus) and brainstems were collected as described in the chick brain atlas [68]. The brain samples were snap-frozen and kept at −80°C until amino acid analysis. After opening the skulls for the brain dissections, the presence of dye in the ventricular system was verified to confirm the correct site of injection. Data for chicks lacking dye were excluded from the data analysis.

### 4.4. Brain Amino Acid Analysis

The free amino acids were analyzed in the diencephalon and brainstem using high-performance liquid chromatography (HPLC), according to the previous methods [69] with slight modifications [70]. The samples were briefly homogenized in ice-cold 0.2 M perchloric acid containing 0.01 mM ethylenediaminetetraacetic acid disodium salt (EDTA.2Na) and left on ice. After 30 min, the homogenates were centrifuged at 20,000× *g* for 15 min at 4 °C. After centrifugation, the supernatants were collected and filtered through 0.2 µm hydrophilic polytetrafluoroethylene filters (Millipore, Bedford, MA, USA). L-amino acid solutions (type ANII, type B, L-asparagine, L-glutamine, and L-tryptophan; Wako, Osaka, Japan) were used to prepare 200 pmol/µL of the standard solution. The standard solutions (10 µL) and brain tissue filtrate (20 µL) were adjusted to a pH of 7 with 1 M sodium hydroxide and dried under reduced pressure (Centrifugal Evaporator, CVE-3000, EYELA, Tokyo, Japan). Then, 10 µL of 1 mol/L sodium acetate-methanol-triethylamine (2:2:1) were used to dissolve dried residues, and the samples were re-dried under reduced pressure, followed by adding 20 µL of methanol-distilled water-triethylamine-phenylisothiocyanate (7:1:1:1). The samples were incubated for 20 min at room temperature to form phenyl thiocarbamoyl derivatives. The standard and samples were subjected to drying, again under reduced pressure, before dissolving in 200 µL of Pico-Tag Diluent (Waters, Milford, CT, USA), and a 0.20 μm filter (Millipore, Bedford, MA, USA) was used to obtain the filtrate. A Waters HPLC system (Pico-Tag free amino acid analysis column (3.9 mm × 300 mm), an Alliance e2695 Separations Module and a Waters 2487 dual-wavelength UV detector and Empower software) was used to apply the solution containing the derivatives. A gradient linear elution program (0%, 3%, 6%, 9%, 40% and 100%) was applied using mobile phase A and B at a flow rate of 1 mL/min at 46 °C. Mobile phase A consisted of 70 mmol/L of sodium acetate trihydrate and acetonitrile at a ratio of 975:25. The sodium acetate solution was adjusted to a pH of 6.45 by adding 10% acetic acid and was then filtered through a 0.45 µm MCE membrane (MF-Millipore, Merck Millipore Ltd., Cork, Ireland). Mobile phase B consisted of water, acetonitrile and methanol at a ratio of 40:45:15. The UV wavelength was set at 254 nm to determine the concentrations of amino acids. The concentrations of amino acids in the brain samples were expressed as pmol/mg, wet tissue. The system did not distinguish between L- and D-isomers; thus, only names of amino acids are provided.

### 4.5. Statistical Analyses

The rectal temperature results were analyzed using a repeated measure three-way analysis of variance (ANOVA), where the main effects were taurine, CRF and time, followed by the Tukey–Kramer test as a post hoc analysis. A two-way ANOVA was carried out for distress vocalizations, behavioral results and amino acids, where the main effects were taurine and CRF, followed by the Tukey–Kramer test as a post hoc analysis when a significant interaction was detected. The differences were considered significant at *p* < 0.05. The data are presented as means ± standard error of the mean (SEM). The Thompson rejection test was applied to eliminate the experimental data that contained outliers (*p* < 0.01) [71]. The statistical analysis was performed using StatView version 5.0 (SAS Institute Inc., Cary, NC, USA).

## Figures and Tables

**Table 1:** Effect of the intracerebroventricular injection of taurine or CRF on the amino acid concentrations in the diencephalon of chicks exposed to social isolation stress.

Free Amino Acids	Saline			Taurine			CRF			Taurine + CRF			*p* Value		
													Taurine	CRF	Taurine × CRF
*Essential amino acids*															
Leucine	221	±	6	198	±	7	212	±	7	208	±	2	*p* < 0.05	NS	NS
Isoleucine	115	±	5	97	±	4	113	±	6	100	±	4	*p* < 0.005	NS	NS
*Nonessential amino acids*															
Taurine	4183	±	57	5863	±	162	4232	±	101	5276	±	263	*p* < 0.0001	NS	NS
Tyrosine	105	±	5	101	±	4	121	±	7	102	±	5	*p* < 0.05	NS	NS
Glutamic acid	6650	±	69	6308	±	68	6592	±	144	6246	±	119	*p* < 0.005	NS	NS
Asparagine	374	±	7	349	±	10	383	±	7	360	±	11	*p* < 0.05	NS	NS
Alanine	893	±	15	860	±	21	897	±	29	838	±	9	*p* < 0.05	NS	NS
β-Alanine	319	±	16	298	±	12	318	±	8	286	±	11	*p* < 0.05	NS	NS
Cystathionine	16	±	1	15	±	1	16	±	1	14	±	1	*p* < 0.05	NS	NS
3-Methylhistidine	304	±	32	277	±	25	323	±	21	238	±	19	*p* < 0.05	NS	NS

The number of chicks used in each group was 7−9. Values are means ± SEM in pmol/mg wet tissue. NS, not significant; CRF, corticotropin-releasing factor.

**Table 2:** Effect of the intracerebroventricular injection of taurine or CRF on amino acid concentrations in the brainstem of chicks exposed to social isolation stress.

Free Amino Acids	Saline			Taurine			CRF			Taurine + CRF			*p* Value		
													Taurine	CRF	Taurine × CRF
*Essential amino acids*															
Isoleucine	93	±	4	89	±	2	95	±	2	84	±	4	*p* < 0.05	NS	NS
Phenylalanine	88	±	4	84	±	6	95	±	6	75	±	2	*p* < 0.05	NS	NS
Glycine	2814	±	66	2955	±	62	2953	±	63	3067	±	44	*p* < 0.05	*p* < 0.05	NS
*Nonessential amino acids*															
Taurine	2686	±	66 ^b^	3903	±	253 ^a^	2982	±	127 ^b^	3168	±	90 ^b^	*p* < 0.0005	NS	*p* < 0.005
Tyrosine	86	±	5 ^ab^	88	±	4 ^ab^	107	±	8 ^a^	81	±	4 ^b^	*p* < 0.05	NS	*p* < 0.05
Cysteine	100	±	5	88	±	4	93	±	5	86	±	5	*p* < 0.05	NS	NS

The number of chicks used in each group was 7−9. Values are means ± SEM in pmol/mg wet tissue. Different superscripts in the same row indicate significant differences (*p* < 0.05) between saline, taurine, CRF and CRF + taurine groups. NS, not significant; CRF, corticotropin-releasing factor.

## Data Availability

Data are contained within the article.

## Supplementary Materials

The following are available online at https://www.mdpi.com/article/10.3390/metabo12010083/s1, Table S1: Effect of an intracerebroventricular injection of taurine or CRF on amino acid concentrations in the diencephalon of chicks exposed to social isolation stress; Table S2: Effect of an intracerebroventricular injection of taurine or CRF on amino acid concentrations in the brainstem of chicks exposed to social isolation stress.
