# Supplementary material for: Central Taurine Attenuates Hyperthermia and Isolation Stress Behaviors Augmented by Corticotropin-Releasing Factor with Modifying Brain Amino Acid Metabolism in Neonatal Chicks
Source: Metabolites. 2022 Jan 16;12(1):83. doi: 10.3390/metabo12010083 (PMC8781603; doi:10.3390/metabo12010083)
Supplement: Supplementary file 1 [file metabolites-12-00083-s001.zip › metabolites-1542022-supplementary.pdf]

Table S1. Effect of an intracerebroventricular injection of taurine or CRF on amino acid concentrations in the diencephalon of chicks exposed to social isolation stress.

Free amino acids	Saline			Taurine			CRF			Taurine + CRF			P value		
													Taurine	CRF	Taurine × CRF
Essential amino acids															
Arginine	283	±	15	255	±	11	271	±	18	282	±	11	NS	NS	NS
Glycine	1448	±	28	1486	±	38	1504	±	33	1458	±	22	NS	NS	NS
Histidine	181	±	6	185	±	6	177	±	6	185	±	10	NS	NS	NS
Methionine	262	±	43	199	±	34	260	±	40	229	±	48	NS	NS	NS
Phenylalanine	106	±	7	108	±	13	105	±	8	95	±	7	NS	NS	NS
Threonine	651	±	13	653	±	45	675	±	30	601	±	29	NS	NS	NS
Tryptophan	35	±	2	38	±	7	36	±	2	31	±	3	NS	NS	NS
Valine	187	±	9	179	±	7	163	±	7	165	±	7	NS	$P<0.05$	NS
Nonessential amino acids															
α-Aminoadipic acid	314	±	9	312	±	7	317	±	7	300	±	7	NS	NS	NS
Aspartic acid	2770	±	21	2776	±	39	2693	±	25	2625	±	49	NS	$P<0.005$	NS
Citrulline	319	±	13	318	±	10	314	±	9	298	±	12	NS	NS	NS
Cysteine	131	±	11	121	±	7	126	±	8	118	±	5	NS	NS	NS
GABA	5308	±	91	5287	±	109	5167	±	71	5195	±	82	NS	NS	NS
Glutamine	12276	±	251	11399	±	284	11859	±	237	11932	±	223	NS	NS	NS
Hydroxyproline	137	±	8	119	±	3	139	±	8	142	±	9	NS	NS	NS
1-Methylhistidine	601	±	67	514	±	42	548	±	52	470	±	28	NS	NS	NS
Ornithine	33	±	1	37	±	6	32	±	3	29	±	2	NS	NS	NS
Proline	371	±	19	337	±	13	352	±	20	329	±	11	NS	NS	NS
Serine	949	±	19	897	±	33	950	±	18	933	±	32	NS	NS	NS

The number of chicks used in each group was 7–9. Values are means ± S.E.M. Values are means ± SEM in pmol/mg wet tissue. GABA, γ-amino butyric acid; NS, not significant; CRF, corticotropin-releasing factor.

Table S2. Effect of an intracerebroventricular injection of taurine or CRF on amino acid concentrations in the brain stem of chicks exposed to social isolation stress.

Free amino acids	Saline			Taurine			CRF			Taurine + CRF			P value		
													Taurine	CRF	Taurine × CRF
Essential amino acids															
Arginine	294	±	20	273	±	14	275	±	22	309	±	6	NS	NS	NS
Histidine	141	±	7	149	±	5	149	±	5	160	±	10	NS	NS	NS
Leucine	166	±	6	163	±	4	163	±	6	161	±	5	NS	NS	NS
Methionine	334	±	68	309	±	57	294	±	63	320	±	70	NS	NS	NS
Threonine	567	±	20	630	±	33	616	±	32	611	±	37	NS	NS	NS
Tryptophan	13	±	1	20	±	6	22	±	7	30	±	14	NS	NS	NS
Valine	170	±	6	170	±	4	156	±	3	155	±	8	NS	$P<0.05$	NS
Nonessential amino acids															
Alanine	781	±	13	821	±	22	810	±	19	775	±	21	NS	NS	NS
β-Alanine	246	±	7	230	±	8	236	±	12	246	±	10	NS	NS	NS
α-Aminoadipic acid	347	±	9	319	±	17	321	±	17	354	±	12	NS	NS	$P<0.05$
Asparagine	320	±	14	313	±	11	317	±	12	323	±	13	NS	NS	NS
Aspartic acid	2654	±	56	2709	±	30	2644	±	33	2651	±	59	NS	NS	NS
Citrulline	284	±	19	295	±	14	304	±	23	325	±	18	NS	NS	NS
Cystathionine	29	±	8	38	±	6	41	±	7	40	±	8	NS	NS	NS
GABA	3057	±	75	3012	±	106	3080	±	69	3017	±	80	NS	NS	NS
Glutamic acid	5880	±	46	5749	±	107	5920	±	133	5885	±	82	NS	NS	NS
Glutamine	10230	±	940	9765	±	760	9498	±	746	8858	±	733	NS	NS	NS
Hydroxyproline	138	±	7	128	±	4	140	±	7	149	±	8	NS	NS	NS
1-Methylhistidine	440	±	32	409	±	21	388	±	27	438	±	26	NS	NS	NS
3-Methylhistidine	787	±	158	487	±	48	538	±	71	622	±	72	NS	NS	NS
Ornithine	20	±	2	21	±	3	25	±	5	15	±	2	NS	NS	NS
Proline	231	±	20	249	±	13	268	±	23	264	±	21	NS	NS	NS
Serine	778	±	11	782	±	24	826	±	29	815	±	14	NS	NS	NS

The number of chicks used in each group was 7–9. Values are means ± S.E.M. Values are means ± SEM in pmol/mg wet tissue. GABA, γ-amino butyric acid; NS; not significant; CRF, corticotropin-releasing factor.
